# Supplementary material for: 30-day survival and injury severity in traffic accidents in Iran: A mixed-method study protocol
Source: PLoS One. 2025 Dec 18;20(12):e0338841. doi: 10.1371/journal.pone.0338841 (PMC12714263; doi:10.1371/journal.pone.0338841)
Supplement: Attachment — Submitted filename: Response to Reviewers.docx [file pone.0338841.s002.docx]

**Response to Reviewer**

We would like to express our sincere gratitude to the reviewers and editors of PLOS ONE for the time, effort, and thoughtful consideration they have dedicated to evaluating our manuscript. We highly appreciate the constructive comments and suggestions, which have greatly helped us to improve the clarity, rigor, and overall quality of our work. Our responses to the reviewers’ comments have been presented in a table, included in the attached file.

* Thank you for your helpful suggestion. We appreciate the recommendation to deposit our laboratory protocols in protocols.io to enhance reproducibility. The protocol has been registered in protocols.io with DOI: dx.doi.org/10.17504/protocols.io.n2bvjez2xgk5/v1.

| Reviewer #1 | | | |
| --- | --- | --- | --- |
|  | **Review Comments** | **Response to Reviewer** | **line** |
| 1 | The current title is too lengthy and complex. Consider simplifying it to something like: "30-Day Survival and Injury Severity in Traffic Accidents in Iran: A Mixed-Method Study Protocol". | We agree that the original title was lengthy, and we have revised it to a more concise and clear version: “30-Day Survival and Injury Severity in Traffic Accidents in Iran: A Mixed-Methods Study Protocol” | 1-5 |
| 2 | The manuscript inconsistently uses both '2025' and '1404'. It’s recommended to clarify this at the beginning by stating 1404 is equivalent to 2025, and then stick to one format throughout the text. | We have revised the manuscript accordingly, and we believe it has improved the overall quality. | 108, 111, 114, 116, 118, 134, 370 |
| 3 | Ethical approval details are mentioned but not specific (e.g., reference number). | The study was approved by the Ethics Committee of the University of Social Welfare and Rehabilitation Sciences, reference number (IR.USWR.REC.1404.037) 28-05-2025. | 237-238 |
| 4 | The qualitative analysis section does not clearly explain coding, category development, or how trustworthiness will be established. Add detail on data analysis steps, such as coding strategies and verification methods (e.g., member checking, triangulation). | This section has been added to the manuscript "Qualitative content analysis with an inductive approach will be employed to analyze the qualitative data. In the first step, interview transcripts will be prepared verbatim and serve as the primary research data. In the second step, the texts will be divided into semantic units, which will then be summarized and condensed. In the third step, abstracting of semantic units and code selection will be conducted. Both explicit and implicit concepts, drawn from participants’ statements, will be identified in sentences or paragraphs and assigned codes, followed by coding and summarization. In the fourth step, through continuous comparison of similarities, differences, and relationships, codes representing a single concept will be grouped into categories and subcategories. Ambiguous points will be clarified through follow-up interviews with participants, ensuring that all codes are accurately placed within the appropriate categories. In the fifth step, at the interpretive level, categories will be abstracted, central concepts identified, and the main and overarching themes extracted. These concepts will be reviewed to ensure that the internal themes accurately represent the entire dataset".  "In this study, we apply Guba and Lincoln’s (1985) criteria to evaluate trustworthiness, which includes validity, reliability, confirmability, and transferability.  **Validity:** The researcher enhances the credibility of the findings through prolonged engagement, immersion in the data, and verification by both participants and observers.  **Reliability:** All interviews are recorded, transcribed verbatim, carefully documented, and reviewed by experts.  **Confirmability:** Every research step and decision is systematically documented to allow for transparency and future follow-up.  **Transferability:** Detailed descriptions of the participants, research context, and data collection procedures are provided to strengthen the generalizability of the findings". | 319-348 |
| 5 | The Cox model design omits discussion of potential interaction terms (e.g., age × ISS). Consider planning for interaction effect testing in the regression analysis. | In Cox regression, in addition to examining the relationship between individual variables and potential confounders, we also assess the interaction terms of key variables such as age, gender, and injury severity. | 227-229 |
| 6 | Even as a protocol, the study should acknowledge possible limitations such as potential bias, loss to follow-up, or limited generalizability | As this is a study protocol, we have now included a section highlighting possible limitations, including potential bias, loss to follow-up, and limited generalizability. We recognize that these factors may affect the study’s outcomes and interpretation, and we will address them carefully in the final study report. | 379- 383 |
| 7 | If data collection tools such as the researcher-made checklist are central to the study, include an English version as an appendix or describe it in more detail. | The revisions and additions have been made | appendix |
| 8 | While most of the manuscript is formal, some parts are explanatory or informal. A thorough language revision is needed to ensure consistency. | We have carefully revised the manuscript to ensure consistent, formal language throughout and have addressed all instances of informal or explanatory wording. | the full article |
| Reviewer #2 | | | |
|  | **Review Comments** | **Response to Reviewer** | **Page-line** |
| 1 | provide reference for Sample size. | We have now provided a reference to support the determination of our sample size in the manuscript. | 153 |
| 2 | Details of the aims can be the same in everywhere of the protocol. | We agree with your suggestion and have ensured that the details of the study aims are consistently presented throughout all sections of the protocol. | 19-23, 107-123 |
| 3 | Provide diagram of the data collection and study steps as a figure. | we have now provided a diagram illustrating the data collection process and the study steps, as requested. |  |
